# Prevalence of Anemia in Pediatric IBD Patients and Impact on Disease Severity: Results of the Pediatric IBD-Registry CEDATA-GPGE®

**DOI:** 10.1155/2017/8424628

**Published:** 2017-12-05

**Authors:** Jan de Laffolie, Martin W. Laass, Dietmar Scholz, Klaus-Peter Zimmer, Stephan Buderus, CEDATA-GPGE Study Group

**Affiliations:** ^1^General Pediatrics & Neonatology, Justus-Liebig-University, Giessen, Germany; ^2^Department of Pediatrics, Medical Faculty Carl Gustav Carus, Technische Universität Dresden, Dresden, Germany; ^3^Department of Child and Adolescent Psychiatry, Psychotherapy and Psychosomatics, University of Leipzig, Leipzig, Germany; ^4^Pediatric and Adolescent Medicine, GFO Kliniken Bonn, St. Marien-Hospital, Bonn, Germany; ^5^Gesellschaft für pädiatrische Gastroenterologie und Ernährung, Chausseestr 128-129, Berlin, Germany

## Abstract

**Aim:**

To determine the prevalence of anemia and its association with disease severity in children and adolescents with IBD.

**Methods:**

CEDATA-GPGE is a registry for pediatric patients with IBD in Germany and Austria from 90 specialized centers. As markers of disease severity, analysis included patient self-assessment on a Likert scale (1–5; 1 = very good) and physicians' general assessment (0 = no activity to 4 = severe disease) and the disease indices. Anemia was defined as hemoglobin concentration below the 3rd percentile.

**Results:**

Prevalence of anemia was 65.2% in CD and 60.2% in UC. Anemic CD and UC patients showed significantly worse self-assessment than patients without anemia (average ± standard deviation; CD: 3.0 ± 0.9 versus 2.5 ± 0.9, *p* < 0.0001; UC: 2.9 ± 0.9 versus 2.3 ± 0.9, *p* < 0.0001). Accordingly, physicians' general assessment (PGA) was significantly worse in anemic than in nonanemic patients in CD (*p* < 0.0001) and UC (*p* < 0.0001). PCDAI in anemic CD, *p* < 0.0001, and PUCAI in anemic UC patients, *p* < 0.0001, were significantly higher than in nonanemic patients. 40.0% of anemic CD and 47.8% of anemic UC patients received iron during follow-up.

**Conclusion:**

Almost 2/3 of pediatric IBD patients are anemic. Patients' self-assessment and disease severity as determined by PGA and activity indices are worse in anemic patients. Contrastingly, only a minority received iron therapy.

## 1. Introduction

Pediatric onset inflammatory bowel disease (IBD) is a group of chronic, relapsing disorders of the gastrointestinal tract, which affect an increasing number of children and adolescents. While incidence in adult populations seems to stabilize, pediatric onset is increasing. In recent decades, there has been a general increase in the incidence and prevalence of IBD in both industrialized and emerging countries [1]. A shift towards earlier onset of IBD has been observed within pediatrics [2]. Recent studies indicate incidence rates of 5 to 11 per 100,000 children under the age of 18 years [3]. About 20% of all IBD patients are diagnosed before the age of 18 years, and early onset disease is associated with a high degree of patient morbidity, missed work or school, decreased quality of life, and psychosocial issues [4–7]. Anemia is a common problem in IBD patients. In pediatric IBD, anemia is reported in up to 75% of patients [8, 9]. The description of various phenotypic presentations in pediatric IBD and a higher rate of anemic patients has also led to the notion of anemia being an important extraintestinal manifestation of IBD [7, 10].

Anemia can occur in pediatric IBD patients for different reasons: gastrointestinal blood loss, insufficient dietary intake of iron, decreased absorption of dietary iron, and decreased activity of bone marrow because of chronic inflammation [11]. Anemia potentially leads to significant reduction in health-related quality of life, impairs physiological development, growth, and psychosocial processes [12], and poses a significant economic burden on health systems [13].

This interferes with the aims of therapy in pediatric IBD: relief of symptoms, normal growth and pubertal development, improved quality of life, but minimization of drug toxicity [14]. Diagnosis and adequate treatment of iron deficiency anemia is important, especially since previous studies have shown that duration of anemia can be reduced by iron supplementation [15].

Previous studies on anemia in pediatric IBD included relatively small patient numbers [16]. The aim of this study is to provide prevalence data of anemia and iron treatment in the, to date, largest cohort of pediatric IBD patients at time of diagnosis and after one to five years of their disease. We also explored the impact of anemia on subjective and objective measurements of disease activity and subjective general well-being.

## 2. Methods

CEDATA-GPGE is a registry, currently based at the University Children's Hospital Giessen for pediatric IBD patients (0–18 years of age) in German-speaking countries from over 90 pediatric gastroenterology centers in Germany and Austria [7]. The database included all patient data sets with diagnosis from 2004 until July 2010. Inclusion criteria comprised of at least one follow-up within 3 months of the first report to the registry. This period of time (time of diagnosis until the first follow-up) will subsequently be referred to as “at diagnosis.” To further improve data quality, only patients with at least two follow-up visits within one year following diagnosis were included. Data were analyzed retrospectively for the time of diagnosis and during follow-up (mean 103 days, range 1–1645 days).

Anemia was defined as hemoglobin below the 3rd percentile for age and gender in recent population-based pediatric Hb reference values in Germany as published by the KiGGS Study (Table 1). To evaluate the clinical significance of anemia, severe anemia was defined as a concentration of hemoglobin more than 2 g/dl below the 3rd percentile for age and gender [17].

Patient self-assessment was quantified on a Likert scale from 1 to 5 (1 = very good and 5 = very bad). Disease activity was measured by physicians' general assessment on a 4 point scale (1 = no activity/remission and 4 = severe activity) and by calculation of the Pediatric Crohn's Disease Activity Index (PCDAI) for patients with CD and the Pediatric Ulcerative Colitis Activity Index (PUCAI) [18, 19]. The hematocrit in PCDAI and rectal bleeding in PUCAI were excluded, since they are directly related to bleeding and anemia to calculate a modified PCDAI or PUCAI, respectively, at time of diagnosis.

Prevalence of anemia were calculated once at the time of diagnosis and subsequently in a cross section fashion in all patients after one to five years follow-up of their disease course. The registry does not hold data on ferritin or transferrin. A decrease in mean corpuscular volume (MCV) was considered as suggestive for iron deficiency anemia. Since azathioprine can increase MCV, azathioprine therapy was compared in patients with anemia and without anemia to correct for potential bias.

### 2.1. Statistical Methods

Analysis was performed in SAS (Cary, NC, USA) and R (The R Foundation for Statistical Computing, Vers 3.0.2). Some of the graphics and tables were redrawn in Microsoft Excel. Distribution was assessed for normality, and *t*-test was performed when comparing continuous or pseudonormal data; otherwise, Mann–Whitney *U* Test was performed where appropriate. Statistical significance was assumed when *p* < 0.05. The database and reporting procedure were examined and approved by the relevant ethics committees and data protection employees of the three institutions that maintained the registry during the time period covered by the research (the Institute for Medical Informatics and Biometry (IMB) of Technische Universität Dresden; the Institute of Epidemiology at the Helmholtz Zentrum, LMU Munich; and the Department of General Pediatrics and Neonatology, JLU Giessen). The Austrian hospitals that participated also obtained local ethics approval.

## 3. Results

3554 patients were initially included in the study, with a total of 25,765 patient contact documentations. 109 patients were excluded because of incomplete initial data, 51 for missing date of primary diagnosis, 22 for implausible data or typing errors, and 616 patients for missing the second follow-up. 2756 patients (1753 CD, 882 UC, and 121 IBD-U (IBD unclassified)) were available for analysis.

Of 2756 children and adolescents with IBD, 1743 (63.2%) suffered from anemia at any time during the disease course—referred to standard values for hemoglobin from the KiGGS study (Table 1). Anemia was slightly more frequent in CD (65.2%) than in UC (60.2%, *p* = 0.013) and in IBD-U (61.8%, *p* = 0.049).

**Figure 1:**
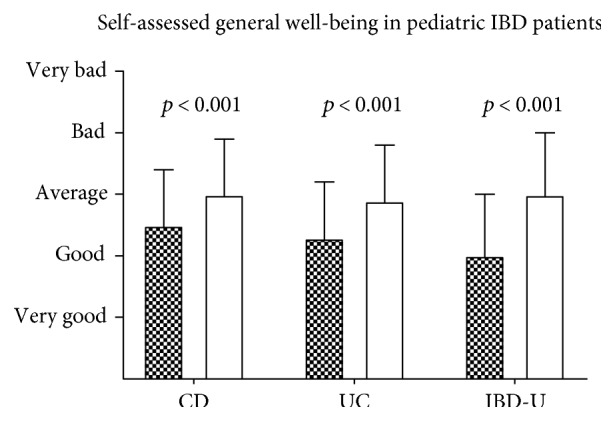
Self-assessment of general well-being of pediatric IBD patients comparing anemic (white) and nonanemic (filled bars) patients (mean ± SD where parametric, median interquartile range (IQR) where nonparametric) (*n* = CD 1144/609; UC 531/351; CI 68/53).

Anemic CD patients assessed their own well-being significantly worse than CD patients without anemia (3.0 ± 0.9 versus 2.5 ± 0.9, mean ± standard deviation, *p* < 0.0001). Similar significant differences were found in anemic versus nonanemic UC patients (2.9 ± 0.9 versus 2.3 ± 0.9, mean ± standard deviation (SD), *p* < 0.0001) and in anemic versus nonanemic IBD-U (median 3.0, interquartile range (IQR) versus median 2.0, IQR; *p* < 0.001 Mann–Whitney *U* test) (Figure 1).

**Figure 2:**
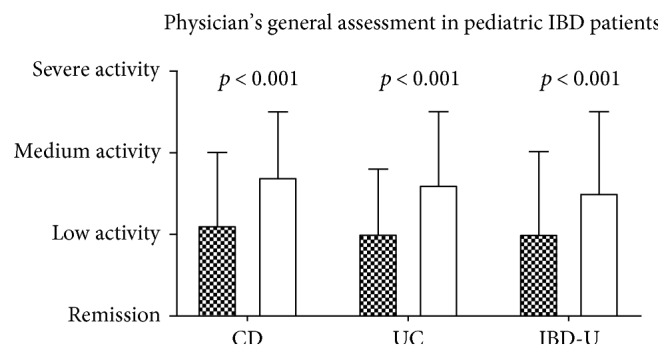
Physicians' general assessment of disease activity in anemic (white) versus nonanemic (filled bars) patients sorted by diagnosis (*n* = CD 1144/609; UC 531/351; CI 68/53).

The physicians' general assessment was significantly worse in anemic than in nonanemic patients in CD (2.7 ± 0.8 versus 2.1 ± 0.9, *p* < 0.0001), UC (2.6 ± 0.9 versus 2.0 ± 0.8, *p* < 0.0001), and IBD-U (median 2.5 versus 2.0, IQR 1; *p* = 0.0003 MWU). In all three disease groups, anemic patients had higher disease activity with a median between low and medium activity (Figure 2).

Anemic CD patients showed a significantly higher PCDAI (27.1 ± 14.1 versus 18.7 ± 12.0, mean ± SD, *p* < 0.0001) than nonanemic, PUCAI was significantly higher in anemic versus nonanemic UC patients (36.1 ± 24.7 versus 19.1 ± 20.4, *p* < 0.0001).

The Pearson correlation coefficient for disease activity indices (PCDAI or PUCAI, resp.) and hemoglobin concentration in CD patients and UC patients was moderately negative with −0.66 and −0.45 (*p* = 0.0057; *p* = 0.0173).

**Figure 3:**
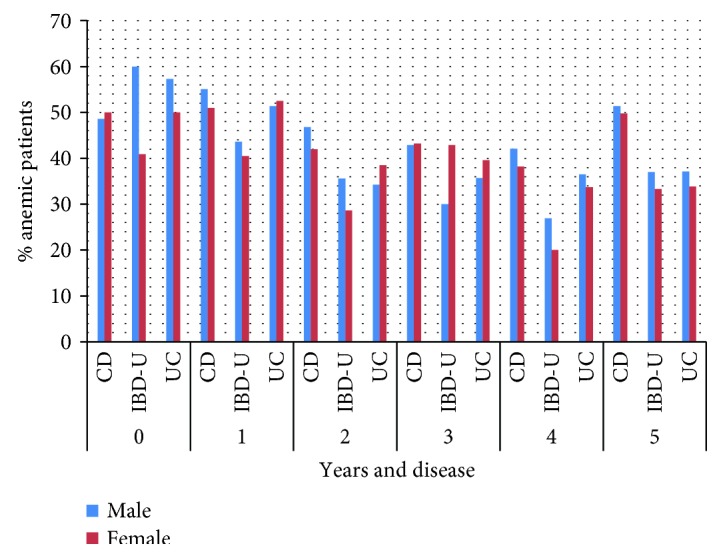
Prevalence of anemia in the first five years of disease by disease and gender (*n* at time points are: 0 = 654; 1 = 1664, 2 = 1559; 3 = 1293; 4 = 1022; 5 = 1114).

The prevalence of anemia during follow-up in the three IBD groups for the first 5 years is displayed in Figure 3. Noticeable is the decrease in prevalence of anemia in UC during the first five years of disease in comparison to a relatively unchanged high prevalence in CD.

Average MCV was significantly below normal in all patient groups (see Tables 2 and 3 reference values from KiGGS study). MCV was significantly lower in patients without azathioprine than in patients with azathioprine (regardless of anemia): CD (*n* = 956 with azathioprine versus 798 without azathioprine), 77.29 ± 7.54 versus 81.67+ −7.76; UC (*n* = 550 versus 331), 80.06 ± 7.53 versus 84.24 ± 7.76; and IBD-U (*n* = 90 versus 31), 77.27 fl ± 7.51 versus 84.27 fl ± 6.28, *p* < 0.0001 for all). 60.1% of patients on azathioprine and 65.3% without azathioprine presented with anemia (*p* = 0.003, OR 0.79 (0.67–0.92 95% CI)). While 38% of anemic patients on azathioprine had low MCV, the share in patients without azathioprine was 56% (*p* < 0.001, OR 0.48 (0.39–0.59)). When correcting for an estimated +4 fl effect of azathioprine on MCV, this difference was not significant (57% versus 56%), so the residual share of microcytic anemia, a correlate of iron deficiency anemia, can be estimated at about 50%. The correlation between MCV and azathioprine dosage was weak to moderate depending on group selection (*r* = 0.14–0.30).

A subgroup analysis was performed for potential correlation of anemia with severity of disease and age group. In the youngest age group (patients < 6 years of age), 46% of CD, 53% of UC, and 38% of IBD-U patients were anemic.

In the group 6 to <10 years, 60% of CD patients, 61% UC patients, and 42% of IBD-U patients showed anemia. In the oldest age group ≥ 10 years, CD patients were anemic in 67% of the cases, UC in 61%, and IBD-U in 58%. Anemia prevalence was significantly higher in adolescents with CD in comparison to CD patients < 6 years (*p* = 0.0005, OR 2.34 (95% CI 1.45–3.79)), and all other differences were not significant.

Of all anemic pediatric IBD patients, 94% had mild and 6% severe anemia. In all age groups, mild anemia was noted in 63.5% of CD patients, 54.6% of UC patients, and 46.6% of IBD-U patients and severe anemia in 2.1%, 5.8%, and 5.3%, respectively. In the patients < 6 years of age, 42.3% of CD patients suffered from mild and 4.2% from severe anemia, while 41.4% of UC patients had mild and 12.1% severe anemia (IBD-U 33.3% and 4.7%).

56.9% of CD patients from the middle age group (6 to <10 years) suffered from mild anemia, 3.6% from severe, and in UC, the percentage was 54.4% and 6.4% (IBD-U 38.4% and 3.8%). The adolescents ≥ 10 years of age had a mild anemia in 65.3% and severe in 1.8% when CD was the diagnosis, 55.7% and 5.1% when UC was the diagnosis, and 52.4% and 5.9% when IBD-U was the diagnosis, respectively.

Only 740 (42.5%) of 1743 anemic patients received iron therapy at the time anemia was present and documented. While in UC, a larger population of female anemic patients received iron supplementation compared to their male counterparts (52.2% (145) versus 43.1% (109), *p* = 0.0365), and this trend did not reach statistical significance in CD or IBD-U (43% (196) versus 38.1% (262); 46.2% (12) versus 38.1% (16)). Data on route of iron supplementation (oral or parenteral) or dosing regiments were not recorded in the registry. CD and UC patients with severe anemia received iron supplementation more often than patients with mild anemia: 41% versus 18.9% of UC patients, 22.2% versus 14.4% of CD patients and 20.4% versus 20% of IBD-U patients.

## 4. Discussion

We found in our study that almost two-thirds of pediatric IBD patients were anemic at the time of diagnosis. So far, there are very few studies that aim at anemia and iron deficiency in pediatric IBD, most of them in rather small cohorts and with short follow-up [8, 9, 15, 16].

Previously reported data from smaller studies have shown similar prevalence of anemia with 70% in children and 42% in adolescents compared to 40% in adults [9, 16].

At presentation, we found a relatively high percentage of anemic patients (all IBD 63.2%, CD 65.5%, UC 60.4%, and IBD-U 51.9%), but less than in other studies reported like the 75% observed by Wiskin et al. [8].

When comparing age groups 0 to <6 years, 6 to <10 years, and ≥10 years, we found an increase in anemia rate at presentation with age (CD 46.5% versus 60.5% versus 67.1%; UC 53.4% versus 60.8% versus 61.0%; IBD-U 38.1% versus 42.3% versus 58.3%). However, the proportion of severe anemic patients decreases (CD 4.2% versus 3.6% versus 1.8%; UC 12.1% versus 6.4% versus 5.2%; IBD-U 4.8% versus 3.8% versus 5.9%). A potential explanation could be the prolonged diagnostic latency in early and very early onset IBD that has been described previously for the German population [20].

Wiskin et al. also reported that 30% of patients with pediatric IBD are anemic two years after their initial diagnosis [8]. In our population, prevalence of anemia two years after the initial diagnosis was higher for CD with 44.8%, but comparable for UC (36.31%) and IBD-U (33.3%). This confirms the relatively high rates found by Gerasimidis et al. with 65% after one year [16].

Looking at the long-term data, in CD patients the anemia rate remains surprisingly stable at about 50%, while prevalence decreases for UC (from 53.4% at diagnosis to 37.9% after 5 years) and IBD-U (from 50% at diagnosis to 35.4% after 5 years).

The impact of anemia on adult patients' quality of life has previously been described. Typical symptoms include not only headache, fatigue, and dizziness but also impairment of cognitive function, stomatitis, and esophageal webs [21]. Our data support the suggested association of anemia with low self-assessed well-being, high disease activity as evaluated by the physician, and higher activity indices, despite correction for obviously anemia-related items, namely, hematocrit in PCDAI and bloody stools and hemoglobin in PUCAI. These items were eliminated in the calculation to avoid a bias towards anemic patients having more inflammatory activity.

### 4.1. Iron Deficiency Anemia

Besides the multifactorial etiology of anemia in IBD, the vast majority of anemic IBD patients suffer from iron deficiency anemia [11, 21].

Wiskin et al. reported prevalences of iron deficiency in 90%, respectively, 95% and 70% in CD and UC patients, respectively, 65% at follow-up after two years [8].

Our data can only provide indirect information on the etiology of anemia from hemoglobin and MCV, since no parameters of iron metabolism like ferritin, transferrin saturation, or soluble transferrin receptor were obtained by the registry.

Decreased MCV as a surrogate marker for iron deficiency anemia has to be interpreted with caution since medication used in pediatric IBD can significantly change MCV. Several studies have suggested a correlation with 6-thioguaninnucleotide (6-TGN) levels, and some even suggested MCV as a surrogate marker instead of measuring metabolites for therapeutic drug monitoring [22].

From our data, correction for azathioprine exposure was difficult to estimate, since metabolism differs significantly between individuals even receiving the same dose of azathioprine or 6-MP [23] and metabolite concentrations were not collected in the registry.

In our patient cohort, we confirmed a significantly higher MCV in the patient groups with azathioprine compared to patients without, but the difference was less pronounced (+4 fl; data not shown). When corrected for this difference, the residual share of microcytic anemia, therefore estimated share of iron deficiency anemia was around 50%.

### 4.2. Iron Therapy

Only about 1/3 to half of anemic patients received iron supplementation (Table 4). This is astonishing since data in adult anemic IBD patients show that treatment is safe and well tolerated in most IBD patients either orally or intravenously and is associated with rapid improvement in clinical, hematological, and quality of life evaluations [24, 25]. Such data does not exist for pediatric patients, but Goodhand et al. also reported that less children (13%) and adolescents (30%) with anemia and IBD received oral iron compared to adults (48%) [9].

Pels et al. showed that expectant management of anemia in pediatric IBD patient leads to a slower hematologic recovery [15]. Persistent anemia in IBD patients is associated with fatigue and other impairments of health-related quality of life [26]. In this study, for the first time shown in a large cohort of pediatric IBD patients, general well-being, as measured by self-assessment, is significantly lower in anemic patients.

Treatment with iron supplementation seems mandatory for patients with proven iron deficiency anemia. For physicians from our registry considering all limitations, an undertreatment of highly prevalent microcytic anemia, potential iron deficient anemia has to be suspected. Possible causes are suspected side effects of iron supplementation orally or parenterally or a suspected cause for anemia in chronic inflammation. A possible explanation could be seen in the high number of patients with relatively mild anemia (less than 2 g/dl Hb below 3rd percentile), but even when looking at severe anemic patients alone, the proportion of patients that receive iron supplementation only increases slightly to 41% in UC, 22.2% in CD, and 20.4% in IBD-U.

The strength of this study is the large number of children and adolescents and the number of follow-up data from our pediatric IBD registry since well-powered studies and registry analysis are scarce in pediatric patients. Limitations are the collection method and study design, which is not tailored to reflect individual courses of disease but rather group characteristics with a cross-sectional instead of longitudinal approach, corresponding to the initial question. To answer questions about treatment and individual patients' benefit, a longitudinal approach has to be taken and is on the way in a future analysis.

## 5. Conclusion

There is a high prevalence of anemia in pediatric patients with IBD. Even more patients with CD are anemic than patients with UC. The rate is highest in adolescents at the onset of disease, and in young children, the rate of patients with severe anemia is higher. Anemia is associated with impairment in patient self-assessment of general well-being, physician assessment of disease activity, and higher disease activity as determined by activity indices. During the disease course, the prevalence of anemia remains high. In contrast, only one-third of patients are treated by iron supplementation. This undertreatment should be addressed by pediatric gastroenterologists.

## Figures and Tables

**Table 1:** Hemoglobin concentration reference values (in g/dl) from the German KiGGS study.

Age (yrs.)	P3	P5	P10	P25	P50 (median)	P75	P90	P95	P97
*Boys (n* = 7205)									
1,5	10,1	10,3	10,6	11,1	11,6	12,1	12,6	12,8	13,0
2	10,3	10,5	10,7	11,2	11,7	12,2	12,7	13,0	13,1
2,5	10,4	10,6	10,9	11,4	11,9	12,4	12,8	13,1	13,2
3	10,5	10,7	11,0	11,5	12,0	12,5	12,9	13,2	13,4
3,5	10,6	10,8	11,1	11,6	12,1	12,6	13,0	13,3	13,5
4	10,8	10,9	11,2	11,7	12,2	12,7	13,1	13,4	13,5
4,5	10,8	11,0	11,3	11,8	12,3	12,8	13,2	13,5	13,6
5	10,9	11,1	11,4	11,8	12,3	12,8	13,3	13,5	13,7
5,5	11,0	11,2	11,5	11,9	12,4	12,9	13,3	13,6	13,8
6	11,1	11,3	11,5	12,0	12,5	13,0	13,4	13,7	13,8
6,5	11,1	11,3	11,6	12,0	12,5	13,0	13,5	13,7	13,9
7	11,2	11,4	11,6	12,1	12,6	13,1	13,5	13,8	13,9
7,5	11,2	11,4	11,7	12,1	12,6	13,1	13,6	13,8	14,0
8	11,3	11,5	11,7	12,2	12,7	13,2	13,6	13,9	14,0
8,5	11,3	11,5	11,8	12,2	12,7	13,2	13,7	13,9	14,1
9	11,4	11,6	11,8	12,3	12,8	13,3	13,7	14,0	14,2
9,5	11,4	11,6	11,9	12,3	12,8	13,3	13,8	14,0	14,2
10	11,5	11,7	11,9	12,4	12,9	13,4	13,8	14,1	14,3
10,5	11,5	11,7	12,0	12,4	13,0	13,5	13,9	14,2	14,4
11	11,6	11,8	12,0	12,5	13,0	13,5	14,0	14,3	14,4
11,5	11,6	11,8	12,1	12,6	13,1	13,6	14,1	14,4	14,5
12	11,7	11,9	12,2	12,7	13,2	13,7	14,2	14,5	14,7
12,5	11,8	12,0	12,3	12,7	13,3	13,8	14,3	14,6	14,8
13	11,9	12,1	12,4	12,9	13,4	14,0	14,5	14,8	15,0
13,5	12,0	12,2	12,5	13,0	13,6	14,2	14,7	15,0	15,3
14	12,1	12,3	12,7	13,2	13,8	14,4	15,0	15,3	15,5
14,5	12,3	12,5	12,8	13,4	14,0	14,6	15,2	15,6	15,8
15	12,5	12,7	13,0	13,6	14,2	14,9	15,5	15,8	16,0
15,5	12,7	12,9	13,3	13,8	14,5	15,1	15,7	16,1	16,3
16	12,9	13,1	13,5	14,0	14,7	15,3	15,9	16,3	16,5
16,5	13,1	13,3	13,6	14,2	14,9	15,5	16,1	16,5	16,8
17	13,2	13,5	13,8	14,4	15,0	15,7	16,3	16,7	17,0
17,5	13,4	13,6	13,9	14,5	15,2	15,9	16,5	16,9	17,2
*Girls (n* = 6870)									
1,5	10,3	10,5	10,8	11,3	11,9	12,4	12,8	13,1	13,2
2	10,4	10,6	10,9	11,4	11,9	12,4	12,9	13,1	13,3
2,5	10,5	10,7	11,0	11,5	12,0	12,5	12,9	13,2	13,3
3	10,6	10,8	11,1	11,5	12,1	12,5	13,0	13,2	13,4
3,5	10,7	10,8	11,1	11,6	12,1	12,6	13,0	13,3	13,4
4	10,7	10,9	11,2	11,7	12,2	12,7	13,1	13,3	13,5
4,5	10,8	11,0	11,3	11,8	12,3	12,7	13,2	13,4	13,6
5	10,9	11,1	11,4	11,8	12,3	12,8	13,2	13,5	13,6
5,5	11,0	11,2	11,4	11,9	12,4	12,9	13,3	13,5	13,7
6	11,0	11,2	11,5	12,0	12,4	12,9	13,3	13,6	13,7
6,5	11,1	11,3	11,6	12,0	12,5	13,0	13,4	13,6	13,8
7	11,2	11,4	11,6	12,1	12,6	13,0	13,4	13,7	13,8
7,5	11,2	11,4	11,7	12,1	12,6	13,1	13,5	13,7	13,9
8	11,3	11,5	11,7	12,2	12,7	13,1	13,5	13,8	13,9
8,5	11,4	11,5	11,8	12,2	12,7	13,2	13,6	13,8	14,0
9	11,4	11,6	11,9	12,3	12,8	13,2	13,6	13,9	14,0
9,5	11,5	11,6	11,9	12,3	12,8	13,3	13,7	14,0	14,1
10	11,5	11,7	11,9	12,4	12,9	13,4	13,8	14,0	14,2
10,5	11,5	11,7	12,0	12,4	12,9	13,4	13,8	14,1	14,2
11	11,6	11,8	12,0	12,5	13,0	13,5	13,9	14,1	14,3
11,5	11,6	11,8	12,1	12,5	13,0	13,5	14,0	14,2	14,4
12	11,6	11,8	12,1	12,6	13,1	13,6	14,0	14,3	14,4
12,5	11,6	11,8	12,1	12,6	13,1	13,6	14,1	14,3	14,5
13	11,6	11,8	12,1	12,6	13,1	13,6	14,1	14,4	14,5
13,5	11,6	11,8	12,1	12,6	13,1	13,7	14,1	14,4	14,6
14	11,6	11,8	12,1	12,6	13,2	13,7	14,2	14,4	14,6
14,5	11,6	11,8	12,1	12,6	13,2	13,7	14,2	14,5	14,7
15	11,5	11,7	12,1	12,6	13,2	13,7	14,2	14,5	14,7
15,5	11,5	11,7	12,0	12,6	13,2	13,7	14,2	14,5	14,7
16	11,5	11,7	12,0	12,6	13,2	13,7	14,3	14,6	14,8
16,5	11,4	11,6	12,0	12,6	13,2	13,8	14,3	14,6	14,8
17	11,4	11,6	12,0	12,5	13,2	13,8	14,3	14,6	14,8
17,5	1,3	11,6	11,9	12,5	13,1	13,8	14,3	14,6	14,9

**Table 2:** Hematological results of Crohn's disease patients with anemia (mean ± SD). Hb: hemoglobin concentration; MCV: mean corpuscular volume; Hct: hematocrit; m: male; f: female.

Time point (years after diagnosis)		Hb (g/dl)	MCV (fl)	Hct (%)
0/at diagnosis	m (*n* = 122)	10.9 ± 1.3	72.9 ± 6.0	34.7 ± 4.7
	f (*n* = 91)	10.0 ± 1.2	73.0 ± 7.4	32.4 ± 3.5

1	m (*n* = 337)	11.8 ± 1.5	78.7 ± 7.7	36.1 ± 5.3
	f (*n* = 227)	11.2 ± 1.5	78.6 ± 7.8	34.4 ± 3.8

2	m (*n* = 273)	11.7 ± 1.5	79.1 ± 8.0	35.2 ± 2.9
	f (*n* = 177)	11.0 ± 1.5	80.2 ± 8.3	34.5 ± 5.4

3	m (*n* = 211)	11.8 ± 1.2	79.7 ± 8.0	36.0 ± 4.1
	f (*n* = 143)	10.8 ± 1.4	80.0 ± 8.0	34.1 ± 3.2

4	m (*n* = 167)	12.0 ± 1.5	79.6 ± 8.8	36.4 ± 4.1
	f (*n* = 99)	11.0 ± 1.1	78.9 ± 8.3	34.3 ± 4.2

5	m (*n* = 203)	12.5 ± 1.8	81.3 ± 7.8	38.0 ± 5.2
	f (*n* = 137)	11.3 ± 1.4	81.0 ± 9.3	33.2 ± 2.1

**Table 3:** Hematological results of ulcerative colitis patients with anemia (mean ± SD). Hb: hemoglobin concentration; MCV: mean corpuscular volume; Hct: hematocrit; m: male; f: female.

Time (years after diagnosis)		HB (g/dl)	MCV (fl)	Hct (%)
0	m (*n* = 51)	10.1 ± 1.5	75.5 ± 7.7	30.9 ± 5.8
	f (*n* = 45)	9.7 ± 1.5	77.8 ± 6.5	31.8 ± 6.4

1	m (*n* = 129)	11.9 ± 1.7	80.5 ± 7.1	36.2 ± 4.4
	f (*n* = 136)	10.9 ± 1.7	80.5 ± 7.7	34.2 ± 6.0

2	m (*n* = 87)	10.9 ± 1.4	79.2 ± 8.9	34.0 ± 7.2
	f (*n* = 92)	10.3 ± 1.2	80.2 ± 9.0	32.4 ± 7.2

3	m (*n* = 74)	11.6 ± 1.7	80.0 ± 7.0	35.6 ± 4.0
	f (*n* = 84)	10.3 ± 1.3	81.5 ± 8.5	32.3 ± 3.5

4	m (*n* = 58)	11.6 ± 1.5	81.2 ± 7.9	36.1 ± 3.9
	f (*n* = 56)	11.2 ± 1.3	80.6 ± 7.1	32.8 ± 7.2

5	m (*n* = 66)	12.0 ± 2.0	82.6 ± 8.5	38.2 ± 4.1
	f (*n* = 74)	11.4 ± 1.5	82.8 ± 7.9	35.1 ± 3.2

**Table 4:** Treatment of anemic patients with iron supplementation.

Iron treatment	Crohn's disease		Ulcerative colitis		IBD-U	
	Male	Female	Male	Female	Male	Female
Yes	262 (38.1%)	196 (43.0%)	109 (43.1%)	145 (52.2%)	16 (38.1%)	12 (46.2%)
No	426 (61.9%)	260 (57.0%)	144 (56.9%)	133 (47.8%)	26 (61.9%)	14 (53.8%)

## Additional Points

*Core Tip*. About 2/3 of pediatric IBD patients are anemic at the time of diagnosis and during follow-up. Anemia correlates to disease severity and patients' self-assessment. In contrast, only about half of the patients receive iron therapy.
